# A Case Report on Copper Beaten Skull Appearance: A Forgotten Entity

**DOI:** 10.7759/cureus.30760

**Published:** 2022-10-27

**Authors:** Prayas Sarda, Keta Vagha, Sneha Kenjale, Kushagra Singh, Ajinkya Wazurkar, Shubhangi Patil Ganvir, Shreyash Huse, Yash Ghulaxe

**Affiliations:** 1 Medicine and Surgery, Jawaharlal Nehru Medical College, Datta Meghe Institute of Medical Sciences, Wardha, IND; 2 Pediatrics, Jawaharlal Nehru Medical College, Datta Meghe Institute of Medical Sciences, Wardha, IND; 3 Surgery, Jawaharlal Nehru Medical College, Datta Meghe Institute of Medical Sciences, Wardha, IND; 4 Pediatrics, Datta Meghe Institute of Medical Sciences, Wardha, IND; 5 Medicine, Jawaharlal Nehru Medical College, Datta Meghe Institute of Medical Sciences, Wardha, IND; 6 Medicine, Datta Meghe Institute of Medical Sciences, Wardha, IND

**Keywords:** intracranial pressure, fgfr2 gene, craniosynostosis, copper beaten skull, crouzon syndrome

## Abstract

An uncommon autosomal dominant condition known as Crouzon's syndrome causes abnormalities of the skull and face. It accounts for 4.8% of all cases of craniosynostosis and is by far the most prevalent condition among them. The fibroblast growth factor receptor-2 (FGFR-2) gene mutation that leads to early suture line closure is the basis for the development of Crouzon’s syndrome. It appears as a copper-beaten skull on radiographs, which may indicate a disruption of the brain's normal growth due to elevated intracranial pressure. This report describes a case of a four-year-old kid who exhibits the typical symptoms of Crouzon’s syndrome like craniosynostosis, hypertelorism, and flattened nasal bridge. We also make an effort to investigate the connection between Crouzon syndrome and the emergence of a copper-beaten skull and related factors.

## Introduction

The brain grows rapidly during the first two years of life, so the skull must expand to accommodate the expanding brain, which occurs through the presence of flexible sutures [1]. When the growth stops, the sutures ossify and merge, forming the vault of the cranium [2]. Premature fusion of this bone is known as craniosynostosis. On radiographs, craniosynostosis can be seen as a copper-beaten appearance of the calvarium, which is brought on by irregular convolutional gyral patterning on the inner table of the calvarium as a result of the growing brain's continuous pulsatile pressure on the flexible cranium, which is best seen on the anterior aspect of the skull [3]. Crouzon's syndrome is among the various types of craniosynostosis, having a prevalence in the range of 15 to 16 cases per million live births [4]. This syndrome is usually associated with a triad of craniosynostosis, mid-face hypoplasia, and proptosis. The syndrome is associated with the mutation of the fibroblast growth factor receptor 2 (FGFR2) gene. Genetic sequencing has revealed the genetic mutation of the B exon of the messenger ribonucleic acid (mRNA) of the gene [5]. With this background, we report a case of a four-year-old boy who presented to us with an abnormal shape of the head and on examination was found to have turricephaly, mild midfacial hypoplasia, proptosis, copper beaten skull on the head radiograph, and was diagnosed with Crouzon syndrome based on the clinical criteria. Through this report, we aim to educate clinicians on this manifestation when dealing with patients presenting with this syndrome.

## Case presentation

**Figure 1:**
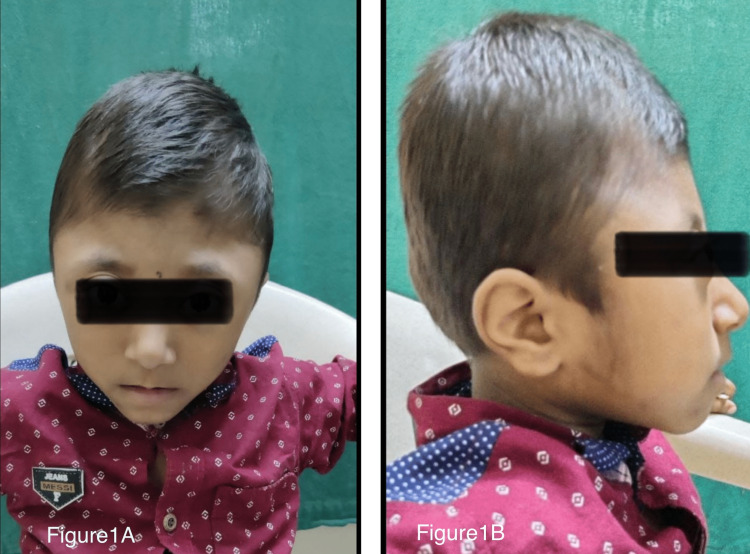
Clinical photographs of the patient A: Seen in the picture is the tower-shaped head i.e., turricephaly secondary to craniosynostosis. Mild mid-facial hypoplasia in the form of a flat nasal bridge and slight proptosis of eyeballs along with hypertelorism can be seen. B: Lateral view where turricephaly can be prominently seen.

A four-year-old boy was brought to us with complaints of a tower-like head noticed at nine months of age. There were no similar features in any of the family members. On clinical examination, the patient had turricephaly, proptosis of both eyes with hypertelorism, flattened nasal bridge, and flat cheekbones attributed to mild mid-facial hypoplasia (Figure 1). His hands and feet were normal i.e., there was no webbing, syndactyly, or polydactyly. He had short stature with a height of 90 cm and a weight of 11 kg. His pulse rate was 88 beats per minute, respiratory rate 24 breaths/minute, SpO2 on room air 98%, and blood pressure 100/60 mmHg. The mental and neurological examination was normal with no other systemic abnormality. The fundus examination suggested mild signs of papilledema in both eyes in the form of a pale optic disc appearance. 

**Figure 2:**
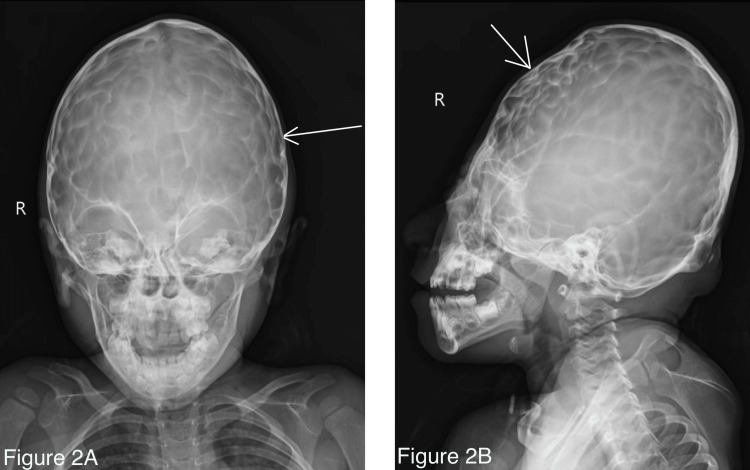
Radiographs of the patient's skull A: Anteroposterior view showing convolutional markings of the gyri suggestive of copper beaten skull appearance. B: Lateral view showing indentations on the skull suggestive of copper beaten skull appearance involving the whole skull secondary to long-standing increased intracranial pressure due to craniosynostosis due to Crouzon syndrome.

His baseline blood investigations showed hemoglobin 10.9 gm/dL, total leukocyte count 5600/cumm, platelets 3.4 lacs/cumm, serum calcium 9.8 mg/dL, serum vitamin D 33 ng/dL, serum phosphate 4.2 mg/dL (Table 1). Radiographs of the head showed a tower-like appearance of the head and prominent convolutional markings on the calvarium suggestive of copper beaten appearance of the skull with mild hypoplasia of the maxilla and the zygoma (Figure 2). The MRI of the brain showed turricephaly with mild dilatation of the right lateral ventricle.

**Table 1:** Baseline blood investigation of the patient compared to the normal range

Parameter	Normal Range	Patient's Baseline Blood Findings
Hemoglobin	11-12.9 g/dl	10.9 g/dl
Total Leucocyte Count	4000-11000/cumm	5600/cumm
Platelet Count	1.5-3.5 lacs/cumm	3.4 lacs/cumm
Serum Calcium	9-11 mg/dl	9.8 mg/dl
Serum Vitamin D	20-40 ng/dl	33 ng/dl
Serum Phosphate	2.8-4.5 mg/dl	4.2 mg/dl

With these findings, the final diagnosis of Crouzon syndrome with copper beaten skull was established. The neurosurgeon who was consulted on the right lateral ventricle dilatation advised that no active intervention was required as the child did not have any obvious signs of raised intracranial pressure and was comfortable. The patient was followed up after one month and was examined for signs of increased intracranial tension. Parents were counseled regarding the preferred management if in case, the child develops raised intracranial pressure in the future. Informed written and verbal consent was taken from the parents of the child for publication of this case report.

## Discussion

Copper beaten skull is a pathological finding seen in an array of clinical conditions. All these conditions share a common theme, which is abnormal intracranial pressure. The major finding is the appearance of a pattern of depressions over the inner surface of the calvarial table [6]. This can occur via several pathological processes, which include but are not limited to, intracranial tumors, space-occupying lesions, neoplasms, obstructive non-communicating hydrocephalus, and the premature fusion of the skull that is craniosynostosis. Copper beaten skull appearance is a feature seen in Crouzon’s syndrome which occurs because of craniosynostosis. Even after this, the juvenile brain continues to grow and because of this growth, there is consistent pressure on the soft, malleable cranial bones. This causes the appearance of multiple markings exhibiting the impressions formed by the gyri and sulci of the surface of the forebrain [7]. These markings are predominantly seen on the interior surface of the calvarium and give the impression of a copper beaten vessel on radiographic imaging. The possible complications that can be seen in such patients could be mental retardation due to increased intracranial pressure, hydrocephalus, chronic tonsillar herniation, and stenosis of the jugular foramen [8].

Copper beaten skull appearance on radiographs encompasses a broad spectrum of conditions. A case report by Bogdanović et al., had the typical copper beaten skull appearance due to a chronic increase in intracranial pressure following a remote head injury in a 19-year-old female patient. She was diagnosed with a case of chronic hydrocephalus which was the cause of the elevated intracranial pressure but the patient couldn't survive [9]. Whereas in another report by Ittyachen et al., the cause of the copper beaten skull couldn't be determined and was an incidental finding: a 24-year-old man with a two-week history of cough, fever and rhinitis and no history suggesting raised intracranial pressure or craniosynostosis. He was treated on an outpatient basis and became asymptomatic within one week of follow-up [10]. This report by Ittyachen et al. shows that the copper beaten skull appearance can occur due to various manifestations of different pathologies. Also, the associated findings tend to differ in different pathologies. For instance, in a case report by Phore et al., a five-year-old female patient presented with carious upper teeth and had a high palatal arch with crowding of teeth, and on radiograph was shown to have copper beaten skull [11].

In our patient, the characteristic copper beaten skull appearance (Figure 2) was seen in the X-ray which was due to the premature fusion of skull sutures at an early age. The patient had all the typical syndromic manifestations of Crouzon's syndrome. However, there was no crowding of teeth and a high-arched palate. Since there was no cognitive impairment in the patient owing to normal intracranial pressure, no active intervention was advised. A similar case reported by Poonia et al., of a five-year-old boy with similar clinical features, had a normal fundus examination along with the absence of developmental delay. As his intracranial tension was normal, the patient was only closely followed up [6]. 

Other syndromic craniosynostoses also tend to present features mimicking copper beaten skull appearance on radiography. It includes the Antler-Bixler syndrome, Apert syndrome which is often associated with manifestations such as syndactyly, Baller-Gerold syndrome, Beare Stevenson syndrome, Carpenter syndrome, Pfeiffer syndrome which may be seen with broad big toes with or without syndactyly, Muenke syndrome, and Saethre-Chotzen syndrome [12]. Treatment of craniosynostosis leading to manifestations including copper beaten skull needs a multidisciplinary approach and early diagnosis and onset of intervention. In severe cases, repositioning and physiotherapy (RPPT) can be considered, however, there is no adequate data on the same [13]. During the first year, surgical methods such as craniotomy are preferred to manage raised intracranial tension, as seen in a case reported by Hlongwa, where a seven-year-old boy who had Crouzon’s syndrome was further planned for a final surgery at 18 years of age for ocular adjustments and reshaping of his head [12]. Maxillofacial surgeries and appropriate procedures can also be done to allow required volume expansion and correct proptosis. Apart from that, a multistage procedure addressing all the anomalies at the same time is also successful in some patients. A seven-year-old girl with Crouzon’s syndrome and raised intracranial tension was treated with a monoblock advancement procedure, which opened the closed sutures while advancing the hypoplastic maxilla and orbits [7].

## Conclusions

In this case, a four-year-old boy presented with a tower-like skull, hypertelorism, and proptosis. Skull radiograph showed copper beaten skull appearance and along with the clinical findings it points towards the diagnosis of Crouzon's syndrome. It is a rare syndrome with unusual findings. The etiology is mainly genetic and the most accepted mechanism is premature craniosynostosis.

Crouzon's syndrome and other premature craniosynostosis syndromes can cause a debilitating effect on the physical and mental development of a child and can potentially decrease the overall quality of the life. This patient would require appropriate medical and surgical management in the future if complications of raised intracranial pressure develop. Only observation and follow-up are necessary as there are no symptoms of elevated intracranial pressure.
